# The Philosophical and Scientific Metaphysics of David Bohm

**DOI:** 10.3390/e20070493

**Published:** 2018-06-26

**Authors:** William Seager

**Affiliations:** Department of Philosophy, University of Toronto Scarborough, Scarborough, ON M1C 1A4, Canada; bill.seager@utoronto.ca; Tel.: +1-416-287-7151

**Keywords:** interpretations of quantum mechanics, David Bohm, mind–body problem, quantum holism

## Abstract

Although David Bohm’s interpretation of quantum mechanics is sometimes thought to be a kind of regression towards classical thinking, it is in fact an extremely radical metaphysics of nature. The view goes far beyond the familiar but perennially peculiar non-locality and entanglement of quantum systems. In this paper, a philosophical exploration, I examine three core features of Bohm’s metaphysical views, which have been both supported by features of quantum mechanics and integrated into a comprehensive system. These are the holistic nature of the world, the role of a unique kind of information as the ontological basis of the world, and the integration of mentality into this basis as an essential and irreducible aspect of it.

David Bohm is famous for re-invigorating and developing the “pilot wave” interpretation of quantum mechanics (QM) originally articulated in 1926 by Louis de Broglie. Bohm’s theory envisages a world of particles which all have definite momenta and positions, albeit the values of which are generally inaccessible. (It is important to point out that the concept of momentum in Bohm’s theory is not straightforward. The fact that we can assign a value of mv to a particle is not directly related to what we would find if we performed a QM measurement of momentum [1,2].) The particles are deterministically “steered” or “guided” by a universal field which is described by the quantum wave function. It is sometimes said that Bohm’s view is a return to a classical picture of the world, embracing atomistic particularity and determinism. For example, the philosopher David Albert forthrightly claims that “the metaphysics of [Bohm’s] theory is exactly the same as the metaphysics of classical mechanics” ([3], p. 174). A recent text book casually characterizes Bohm’s account as one endorsing “local realism” ([4], p. 65). Christopher Fuchs once wrote that “Bohmism” represents a hopeless “return to the womb of classical physics …yuck!” ([5], p. 417).

A core classical theory is of course that of Newton. A “Newtonian world view” is a metaphysical interpretation of a theory which can plausibly be extended to embrace the entire world instead of and speculatively beyond the systems to which it can actually be successfully applied in experiment and technology. The Newtonian viewpoint at issue is that of a world of locally interacting particles which obey well defined laws of nature and whose proclivities for combination lead to all of the complexity of form and the variety of composite systems that we so abundantly observe. As is well known, Newton himself was unable fully to subscribe to Newtonianism in this sense because his theory of gravitation postulated a non-local and instantaneously active “force” generated by every material object which permeated the universe. At the time, the notion of such a thing as “force” was dubious, carrying the taint of the occult (forces are akin to older notions of the “spirit”) and the retrograde Scholastic concept of substantial forms (see [6]). Moreover, Newtonian non-locality is considerably more radical than the more recently discovered quantum variety. It permits (in principle) faster than light, indeed instantaneous, signaling via the mere rearrangement of matter. This extension of the Newtonian metaphysics of nature adds mysterious forces to the push and pull of particle collisions which many at the time regarded as an illicit intrusion of immaterial entities into a part of the world—the material universe—that should be intelligible solely in terms of mechanical principles.

If we take a hard line on Newtonianism—as surrogate for the mechanical metaphysics—then it is hard to seriously maintain that Bohm’s account of quantum mechanics is Newtonian. The quantum potential invoked by Bohm and required to duplicate the empirical success of standard quantum mechanics is irredeemably non-local, and Bohm held views entirely at odds with the mechanical view of the world as consisting of independent, causally interacting individual parts.

Even if we take a softer line, more in line with what Newton himself was willing to postulate, then Bohm’s view is still at odds with a Newtonian picture of the world. There is a split worth noting here in those who work on theories that develop Bohm’s original insight. Bohm himself took the radical and philosophical line I will investigate in this paper. Others, those who develop so-called Bohmian Mechanics, strongly resist any need for a new metaphysical outlook and cleave to a particle-based picture in which the world evolves via, in the words of Peter Holland, “objective processes” ([7], p. 25), albeit non-local ones; numerous papers by Sheldon Goldstein, Detlef Dürr, and their co-workers would also fall on this side of the split (for an overview, see [8]). Newton was not averse to the postulation of forces in nature. However, such forces come in at least two varieties: local forces that are properties of kinds of material bodies and non-local forces such as gravitation which are suspiciously uncaring about the nature of the bodies giving rise to it. The former he welcomed and hypothesized that they would ultimately explain chemistry: “…many things lead me to have a suspicion that all phenomena may depend on certain forces by which the particles of bodies, by causes not yet known, either are impelled toward one another and cohere in regular figures, or are repelled from one another and recede” ([9], p. 382–383). The latter were anathema to Newton, who disparaged those who might favour the idea of action at a distance: “I believe no Man who has in philosophical Matters a competent Faculty of thinking can ever fall into it” ([10], p. 102). For many at the time, even the local forces were suspicious. Pure mechanical contact interaction based upon the impenetrability of matter was the “gold standard” for explanations of the natural world. Bohm’s view could hardly be more different than this vision of classical physics.

Of course, Newton was right; the “forces brigade” won the day over pure mechanism, and Newton’s theory funded the development of classical physics. Still, although physicists became inured to the scandal of action at a distance and non-local instantaneous forces, there were regular calls to recast physical theory in terms of local forces smoothly transmitted through space within some kind of genuinely physical medium. This persistent attitude culminated in Maxwell’s field theory of electromagnetism and, later, Einstein’s revolutionary field-based account of gravitation (for a brief history of the field concept, see [11]). QM entanglement apparently introduces an entirely new kind of non-local relation, which was strongly suggestive to Bohm that a similarly new picture of reality was needed to accommodate it.

Bohm’s account of QM introduces some new ideas and a radically different general outlook on nature. However, it does not make any *empirical* difference: Bohmian predictions are identical to those of “standard” QM. It’s worth noting that there have been some controversial attempts to empirically distinguish the views. Bohm’s account assumes that the initial conditions of a system satisfy the quantum equilibrium condition (that is, the probability distribution of the initial positions of the particles is given by ${\left|\psi \right|}^{2}$). It is conceivable that (parts of) the universe do not abide by this condition. It has also been argued that, although Bohmian theory matches QM statistically, it could vary from it in individual cases, and this divergence might not be absolutely impossible to measure. For references and discussion, see Riggs ([12], pp. 142 ff.). Still, the basic empirical equivalence is the view is often called the Bohmian or (de Broglie–Bohm) *interpretation* of QM. It thus joins the ranks of a host of alternative interpretations and turns into a *metaphysics* of nature.

This raises an unavoidable question of what is metaphysical about interpretations of QM and, in general, what distinguishes a metaphysical question from a scientific question about the structure of reality. Looming behind this issue is a more general one that questions the value of engaging in metaphysics at all. Metaphysical skepticism has a long and distinguished history and debates in this area remain vigorous (see, e.g., [13]). In this paper, I will proceed on the assumption that metaphysical speculation is both possible and valuable, at least to the extent that trying to understand the kind of world which our best science suggests we live in is worthwhile, as the lively debate about the meaning of QM suggests. The quest of metaphysics is succinctly expressed by Wilfred Sellars: “to understand how things in the broadest possible sense of the term hang together in the broadest possible sense of the term” ([14], p. 1). This quest goes beyond the purview of theoretical science. Yet delineating the distinctive nature of metaphysical questions is not a straightforward task because modern science is a more or less direct descendant of early thought about the general nature of reality. When, for example, Anaxogoras postulated that “everything is in everything,” that matter is infinitely divisible, and that every portion of it is a mixture of all possible qualities in different proportions, was he doing proto-science or philosophical metaphysics? (Anaxogoras’s views are in fact intriguing, complicated and far from clear; see [15].) At the time, and for long after, there was no such distinction. As the centuries accumulated, many such questions drifted from the metaphysical towards the scientific pole.

A good clue to a metaphysical question is its distance from empirical testability: the more remote from empirical consequences, the “more metaphysical” the question. I should add the usual rider of “in principle” testability. Technical difficulties in constructing appropriate experimental apparatus does not a metaphysics make. There may also be a general demand on the perceived significance of the question: metaphysics is supposed to tackle big questions. However, I don’t see exactly why there can’t be utterly trivial metaphysical questions. Contrary to positivists, this does not mean the question is empty or meaningless.

To give a pointedly philosophical illustration to illustrate how even the most scholastic seeming question can still link to scientific concerns, consider the nature and identity of composite objects. A persistent question in metaphysics is about the ontological status of such entities. The standard example is the contrast between a bulk lump of clay and the statue artistically formed from it. The lump of clay is the material from which the statue is made. The clay is still there after this operation as is the statue. Are they one and the same entity, about which we merely have two different ways of talking? They occupy exactly the same space and move inexorably together wherever they go. Surely they are one. And yet while we can destroy the statue with a hammer, the bulk lump of clay remains. It seems a reasonable principle that, if one can destroy *x* without destroying *y*, then *x* ≠ *y*. The metaphysics of composite objects can get quite hairy (see [16]). One thing is pretty clear, however: it is hard to think of an empirical test which would answer the question whether the statue and lump are one or two. One might suggest a quick test with a scale. If there are two objects here, then we should sum the weight of lump and statue. But no one thinks that composite objects count for weight beyond that of their constituents (and the function relating mass of constituents to mass of the composite, which is not in general simply summation). Empirically speaking, we already know *everything* we could possibly need to know to answer this question.

Although this question has been selected as a paradigm example of a “purely philosophical” worry, the point is that it does link to scientific concerns. The general problem of understanding material composition is ancient but also has modern offshoots, obviously in studies of the chemical bond and solid state physics. A pure reductionist might dismiss the lump and statue question as merely verbal; what is “really real” is simply the atoms arranged thus and so. However, others hold that “more is different” [17] and that composition introduces new physics into the world. Most dramatically, understanding the place of the ordinary objects of everyday experience connects to the effort to show how a “classical world” can be retrieved from its more fundamental quantum mechanical description. Despite the seeming inevitability of rampant superposition of quantum states, we experience a world of stable and determinate composite objects. The modern investigation of decoherence (see [18,19]) has gone a long way towards solving this problem, which has a history of fairly radical suggestions behind it, as in the suspension of physical law required by the orthodox but infamous projection postulate, the idea that consciousness itself somehow intervenes in the measurement process or the idea that the classical realm is somehow independent of the quantum and in some way is what is fundamentally real.

In fact, although it is true to say that metaphysical questions are remote from empirical testability, we see many suggestive connections between them and scientific theorizing. As another example, there is a perennial tension between the view of the world as an unchanging unity (traditionally represented by Parmenides) versus a view that sees in nature a universal and ceaseless dynamism of change (traditionally represented by Heraclitus). At the appropriate (metaphysical) level of analysis, no empirical test will favour either side of this debate. It is true that we seem to experience change, but a feeling of change is not (necessarily) a changing feeling. It is hard not to see a dim foreshadowing of the “block universe” often associated with relativity theory in the Parmenidean view. Modern quantum cosmology deploys a master equation, the Wheeler–DeWitt equation, that seems to forbid change in the universe. Of course, temporal dynamicists (if I can call them that) push back (see, e.g., [20]). No experiment can decide this, the question seems obviously significant, and important and philosophical reflection here is greatly aided and extended by scientific development.

The grandest metaphysical question of all was posed by Leibniz in 1697: Why is there something rather than nothing at all [21]? It is hard to see how to even begin to grapple with this. Leibniz’s sensible answer was that there must be an absolutely necessary ground of being (which he naturally equated with God) for there could not be a chance “eruption” of contingent reality out of nothingness. Even here, modern physics is not entirely disconnected from this problem. In a recent book, Lawrence Krauss [22] outlines how random but statistically inevitable fluctuation in quantum fields could give rise to particle states from the vacuum state. A trenchant review of Krauss’s book by the philosopher (and physicist by degree) David Albert led to a testy exchange in the New York Times, which makes for an amusing read. Albert’s main point (apparently revealing him to be, in Krauss’s words, a “moronic philosopher”) was that the QFT vacuum is not nothing: “Krauss seems to be thinking that these vacuum states amount to the relativistic-quantum-field-theoretical version of there not being any physical stuff at all,” but this has “nothing whatsoever to say on … why there should have been a world in the first place” [23]. Clearly, nothingness is incompatible with the existence of any quantum field state, vacuum or otherwise. Perhaps an analogy is this. If you shut your eyes, you see “nothing,” but it appears to you as a blacked out visual field. Contrast that with your visual sense of what is behind your head. That is a nothing which is not any kind of “blackness” but simply an absence. Metaphysical nothingness is pure absence.

While Albert is obviously right about this, the relation between the vacuum state and various particle states nonetheless provides an interesting perspective in the philosophy of nothingness. In general, scientific development illuminates and, it must be admitted, usually deepens rather than answers metaphysical questions (The situation is thus reminiscent of the following anec dote: In a summary of lectures on electrodynamics delivered at Moscow University by A. A. Blasov, the following sentence was stated: “The purpose of the present course is the deepening and development of difficulties underlying contemporary theory ...” (as reported in the delightful [24], p. 88)). It would be hard to overstate the significance of the transformation in our metaphysical outlook occasioned by the scientific revolution’s mechanistic metaphysics, which replaced the largely Aristotelean theological metaphysics, which dominated thought for more than a millennium.

In all these examples, to a greater or lesser extent, we see how advances in science serve not to eliminate metaphysical questions, but illuminate them and sometimes to reawaken metaphysical options that had faded from view.

Such a metaphysical question, and one that Bohm was deeply concerned with, is whether the universe is primarily a unified whole, as opposed to a collection of ultimate fundamental parts. As noted above, for a long time, the second, mechanical or part-to-whole, view received vast support from the advance of scientific understanding. In recent times, QM has with its discovery and experimental verification of entanglement, revived universal holism. Theoretical advances once again can underpin or weaken philosophical views in the absence of any decisive empirical test.

Therefore, what is philosophically spectacular about the development of quantum mechanics is the possibility that it heralds an equally momentous shift in metaphysical outlook. The still dominant mechanistic metaphysics, which pictures the world as made of independent interacting parts, will be hard to overturn. And for good reason. It has generated vast insights, leading to revolutions in technology that are now generating consequences on a planetary scale. Its philosophical impact has been no less significant. The rise of physicalism or scientific naturalism as the “default” metaphysics can in large measure be traced to its long history of success (see [25]).

The fundamental appeal of the mechanical metaphysics is its promise of maximum intelligibility and conceptual simplicity. Discover the fundamental parts of which everything is composed, and discover the laws which govern how they interact, and you have in principle the key to understanding the entire world. The idea of part–whole intelligibility also seems to be ingrained in the human psyche. When faced with something we don’t understand our natural instinct is to take it apart and “see how it works.” This has without question served us extremely well, probably since before we were fully human. Part–whole intelligibility arises from understanding how the properties of the parts and their interactions determine the property of the whole. Newton nicely codified this procedure, breaking it into “analysis” and “synthesis”:

By this way of Analysis we may proceed from Compounds to Ingredients, and from Motions to the Forces producing them...And the Synthesis consists in assuming the Causes discover’d, and established as Principles, and by them explaining the Phænomena proceeding from them, and proving the Explanations.

([26], Query 31)

Note here that Descartes, and others, had a similarly named distinction but applied it in its standard domain of logic and mathematics. Newton’s use of the notions in the context of material constitution presumably harks back to the alchemical tradition in which Newton was very well versed.

Let us call this still ongoing attempt to understand reality in terms of a construction out of independent components the *Parts Project*. Rooted in common experience of the material world, after the 17th century, science or, as it was then known, natural philosophy, and most especially what became physics and chemistry, was charged with completely vindicating the commonsense vision that the material world has a part–whole structure. The initial, seemingly crystal clear conception of pure mechanism slowly gave way to a picture which permitted interactions governed by novel forces. In the 19th century, fields were added to the ontology of interacting particles. However, the electromagnetic field had material sources and, initially, a special material substrate in which it inhered. Recall that Maxwell devoted considerable energy to developing mechanical models of the electromagnetic field (see [27], pp. 451 ff.; for discussion of Maxwell’s “ontological intent” with regard to these models, see [28], pp. 55 ff.). As for the ether, Maxwell wrote that “there can be no doubt” about the existence of the “luminiferous aether” whose properties “have been found to be precisely those required to explain electromagnetic phenomena” [29]. The Parts Project assimilated these changes without difficulty.

The Parts Project is arguably humanity’s most successful intellectual endeavour. Its effect on the material conditions of life is undeniable, and its associated physicalist metaphysics of an intelligible—albeit rather aloof, cold and comfortless—picture of reality is both comprehensive and possesses a still growing cultural influence.

But there is a spectre haunting this history of success. Leaving aside the instrumental and technological accomplishments, the *metaphysical* dream behind the Parts Project was exploded with the birth of QM. One can find many, often astonished, expressions of this:

…a particle certainly is…not a durable little thing with individuality;

([30], p. 241)

the historical idea…that the material world is…structured by some kind of interacting “elementary systems” is in sharp contradiction [with] quantum mechanics;

([31], p. 88)

quantum phenomena require us to think in a radical new way, a way in which we will have to ultimately give up both the notion of particles and fields.

([32], p. 116)

David Bohm himself expressed the collapse of the Parts Project in strong terms that prefigured his favoured replacement metaphysics:

[The] entire universe must, on a very accurate level, be regarded as a single indivisible unit in which separate parts appear as idealizations permissible only on a classical level of accuracy of description.

([33], p. 167)

Given our discussion of the nature of metaphysical questions above, it is clear that the problem of interpreting QM is a metaphysical problem. This in part explains the reluctance and even distaste some physicists, notoriously, for example, Richard Feynman, have about the interpretation project. However, one cannot really avoid the metaphysical side of things since it forms a kind of backdrop or implicit viewpoint that conditions thought.

The literature on the interpretation of QM is truly vast and comes with a corresponding proliferation of interpretations (Wikipedia currently lists 18). Some interpretations do indeed posit an in principle empirically detectable change in QM (e.g., dynamic collapse theories). We have to say “in principle” because these new theories must duplicate the predictive successes of standard QM, and these are so numerous and so rigorous that alternative theories must put any empirical divergence from QM in hard to reach corners of experimental search space. However, many interpretations do not imply any distinct empirical predictions and can be regarded as providing relatively pure metaphysical pictures of the world which their proponents take to be the deep lesson of QM.

Far from an attempt to return to something like a classical mechanistic world view of independent interacting particles, Bohm’s interpretation is philosophically extremely radical. Three key features of Bohm’s view are especially worth emphasizing:

- holism;
- information;
- mind.

In metaphysics, the main claim of holism is that the whole is prior to, or more fundamental than, the parts (an excellent philosophical discussion and defense of holism can be found in Schaffer [34]; Ismael and Schaffer [35] explore the connection between holism and QM). Thus, it is in absolute contradiction with the mechanistic picture of the whole being determined by the system of interaction of a set of independent parts. Instead, the *parts* are the derivative entities. Bohm sometimes uses the analogy of mathematical projection from higher to lower dimensional spaces. For example, he writes

we may regard each of the “particles” constituting a system as a projection of a “higher-dimensional” reality, rather than as a separate particle, existing together with all the others in a common three-dimensional space.

([36], p. 238)

Since each entangled particle is a projection of a single encompassing higher dimensional whole, it is not surprising that particle properties are correlated. Bohm explicates the Bell correlations in these terms.

However, holism should not be identified with non-locality. All that non-locality shows is the possibility of interaction (of some sort) between spatially separated features of reality. One could imagine a world of particles that are able to “talk” to one another after they have met and established a “special bond.” But once again we see some signs from QM that favour the holistic interpretation. It seems that the distant connection supported by entanglement does not permit the communication of information. This is so even in the case of non-relativistic QM. There is no a priori reason to expect that; if non-relativistic entanglement had predicted superluminal signaling, this would simply be a false prediction of a false theory. This is noted in Haroche and Raimond ([4], p. 65): “Non-relativistic quantum physics is non-local in a way subtle enough not to contradict the inherently relativistic causality principle.” A holistic view makes better sense of this as a global constraint rather than some very peculiar and highly tuned property of individual particles.

Again, the wave function seems to be more fundamental than the particles. Experimenters are free to choose any measurement basis they want (e.g., position vs. momentum) but cannot as it were “mix and match.” Both the lack of a privileged set of properties associated with the parts and the inability to measure “across” bases suggests the priority of the whole. We can so to speak pull out particulate features if we wish but only as permitted by the nature of the wave function.

There is no doubt that Bohm embraced a holistic interpretation on which the universe is “undivided”:

Ultimately, the entire universe (with all its “particles,” including those constituting human beings, their laboratories, observing instruments, etc.) has to be understood as a single undivided whole, in which analysis into separately and independently existent parts has no fundamental status.

([36], p. 221)

Instead of being the signature of ultimate reality, the world dreamt of by the mechanical philosophers, which is more or less the world of everyday experience, dissolves into a shadowy realm: non-fundamental, derivative, and merely approximate. The development of physics has not proven this, but current theories at least strongly hint if not outright suggest that the holistic metaphysics is to be favoured.

The embrace of holism leaves open the question: what is the nature of (holistic) reality? The metaphysical atomism of the mechanical world view had a simple answer to this question, based upon our intuitive familiarity with objects in the everyday world. According to this view, the world is basically material and matter itself is ultimately resolved into impenetrable, movable, independent, but capable of causal interaction, “chunks” (quite analogous to microscopic lego bricks). This “lego world” is essentially what Richard Feynman was talking about in this famous pronouncement:

If, in some cataclysm, all of scientific knowledge were to be destroyed, and only one sentence passed on to the next generations of creatures, what statement would contain the most information in the fewest words? I believe it is the atomic hypothesis… that all things are made of atoms—little particles that move around in perpetual motion, attracting each other when they are a little distance apart, but repelling upon being squeezed into one another.

([37], v. 1, p. 2)

As attractive and as useful as this picture of the world is, another deep lesson of QM is that we do not live in a lego world. The rather disturbing philosophical consequence of this is that we have lost any positive conception of the nature of matter itself. Matter, or “the physical” in general, has disappeared into an obscurity masked by our vast knowledge of how “it” structures experience. The unease this should engender is suppressed by our false impression that ordinary perception reveals, more or less directly, the nature of matter as hard, massy, and space-filling. Both the growing mathematical abstractness of physical theory and the realization that whatever lies behind our experiential contact with the material world is completely unlike the tiny “marbles” envisioned by traditional atomism leads to the insight that theory reveals only structural or relational properties of the world. These properties tell us how things interact without telling us what those things are. Mass is the “resistance” a body has to motion when a given force is applied; force is that which induces motion in mass. A certain pattern of observable effects is codified by theory, but these patterns tell us nothing about the intrinsic nature of what lies behind them. In the early to mid twentieth century, this was frequently noted. In 1927, Bertrand Russell wrote:

Physics is mathematical not because we know so much about the physical world, but because we know so little: it is only its mathematical properties that we can discover.

([38], p. 125)

and this view is echoed by Arthur Eddington:

Physical science consists of purely structural knowledge, so that we know only the structure of the universe which it describes.

([39], p. 142)

Note that the general thesis that the structural relations which science is limited to revealing require an intrinsic base often goes by the name Russellian Monism and is enjoying a current renaissance of interest (see e.g., [40]).

Now, it may not be part of the job of science to dig down into the intrinsic nature of things; maybe all it can and should deliver is this kind of structural knowledge, which is, in principle, within the realm of empirical testability. This leads to the movement in philosophy of science called structuralism (see [41] for discussion) and its radical offspring, ontological structural realism (see [13]). The metaphysical project of investigating the *what-it-is* that is being structured remains. Here, Bohm’s (with Basil Hiley) notion of “active information” may be important (see [42]). Active information is a proprietary concept of Bohm (again, along with Hiley). It is supposed to explicate the relation of the quantum wave function’s “guidance” role to the particles being guided. It must be admitted that the concept is not without some obscurity. As Bohm and Hiley describe it, it is unclear how exactly active information operates in the world, specifically whether it is simply another operative causal feature, and hence another aspect of the relational structure of the empirical world, or whether it is something deeper which is involved in the structuring itself. I wish to explore the latter interpretation.

Bohm and Hiley characterize active information in terms of the original etymology of the word “information”: to *in-form* or to give form to something. This notion goes back at least to Aristotle’s core distinction between form and matter. In our terms, “form” would refer to the structural features of the world: the pattern of interaction and system of spatial-temporal relations described in physical theory. The “matter” in this case is not *material*—the physical as scientifically characterized, but rather whatever it is that makes the structural relations investigated by science into concrete reality. We might say that this “matter” is what “breathes fire into the equations,” to use Stephen Hawking’s famous phrase ([43], p. 174). Bohm and Hiley hold that active information operates “actively to put form into something or to imbue something with form” ([42], p. 35). Most of their examples I regard as merely illustrative (such things as weak radio signals remotely controlling a much stronger flow of energy) for they would, if interpreted literally, just make active information into another element in the causal-structural nexus, albeit one with a distinctive role. I think the notion of active information is more radical than that.

One way to see this is, following Bohm and Hiley, to contrast active information with what they call “Shannon information.” The latter is what is studied in the theory of communication and information. It is a paradigm example of how theory reveals only relational structure. Bohm and Hiley try to point this out with the claim that Shannon information is “for us,” that is, the significance of information carried in some channel (information theory is in essence an analysis of such channels) is a matter of interpretation (see [44,45]). There is nothing intrinsic to a string of bits that makes it about missile guidance as opposed to, say, a Gilligan’s Island rerun. However, active information is, as Hiley sometimes puts it, “for the particle” (see, e.g., [32]). Such information is intrinsically semantic as opposed to the merely syntactic or structural information of standard information theory.

Active information is not local and pervades the universe outside of or “behind” space, ready to “in-form” aspects of the world, in particular those aspects we call *particles* “to accelerate or decelerate” according to its overall content (see [42], p. 37). As Bohm and Hiley discuss this, we see again the ambiguity between positing more structural features of reality versus positing something which underlies the structure which physics investigates. For example, Bohm and Hiley conjecture that particles such as electrons (that is, those particles we take to be elementary) have “a complex and subtle inner structure” ([42], p. 37). Perhaps this structure is simply more of what physics can investigate, and will reduce to another, albeit deeper, system of causal relations holding between entities whose nature remains ultimately mysterious. Or it could be that we should interpret this as an “inner” nature that underpins the system of physical relations rather than being directly part of it. Although it cannot be disputed that Bohm and Hiley frequently encourage the former interpretation of active information, the latter avoids certain basic objections, such as that whether or how active information can involve energy transfer and the back reaction, or rather the lack of same, on this field of information by the in-formed entities (for such worries, see [46]). Regarding the wave function as embodying information also helps to solve the problem of the very high dimensionality of many body systems. Normally, configuration space is conceived of as merely a way to describe a complicated system; however, if we take the wave function ontologically seriously, then we have to grapple with the mismatch between its extremely high dimensionality and the three dimensions of space found in experience. Bohm developed the idea that the high dimensionality was an intrinsic feature of a “pool of [active] information” (though at the time he discussed this in terms of what he called the “implicate order”) (see [36], pp. 236 ff.). This seems plausible insofar as information is inherently multidimensional, with no intuitive constraint to merely three dimensions. This is evident even within the realm of Shannon information, where information is quantified in terms of bits, each capable of two states. A system or pool of information of *n* bits is then organized in a space of *n* dimensions and the information state is a point in this space. The analogy with configuration space is clear, but in the case of information there is no pre-existing intuitive constraint limiting the space to three dimensions. Of course, as we have seen, Bohm did not think that Shannon information was anything like his active information. However, it is no less clear that a pool of intrinsically semantic information would also have an organizational structure of high dimensionality. Bohm thought that, if we regarded the quantum wave function as fundamentally informational, its high dimensionality might seem less mysteriously connected to the world of experience.

Seeing the universe as based upon an underlying field of information might also help with the so-called problem of “empty branches.” This is the worry that, although the wave function evolves throughout its space, the particles are restricted to certain regions (there are no genuine superpositions of particles in Bohm’s view). This can seem a rather arbitrary imposition of reality on a mere portion of the world as described by the wave function. As David Deutsch put it, “pilot-wave theories are parallel-universes theories in a state of chronic denial” ([47], p. 225).

But this misunderstands the nature of the particles within Bohm’s metaphysics. Particles are abstractions of the holistic reality or can be regarded as projections from the higher dimensional underlying reality where we find active information. They are not to be thought of as privileged markers of what is physically real as opposed to the ghostly empty branches of the universal wave function. Of course, thinking in terms of particles can be useful, perhaps even indispensable. As Bohm puts it:

Under the ordinary conditions of our experience, these projections will be close enough to independence so that it will be a good approximation to treat them in the way that we usually do, as a set of separately existing particles all in the same three-dimensional space.

([36], p. 239)

If the introduction of this new sort of intrinsically semantic information into the heart of a world hypothesized to be fundamentally holistic was not strange enough, the final aspect of Bohm’s metaphysical interpretation of QM is more peculiar still. We can approach—gingerly—this feature of Bohm’s philosophy by asking if we are familiar with any source of intrinsic semantic information? Information is everywhere, but sources of information that do not require interpretation are rare. The need for *interpretation* is of course the clue we need. Mental states are the terminus of interpretation and seem to be the only carriers of information which is intrinsically semantic. This suggests a possible connection between active information and mentality. Bohm did indeed try to forge such a connection, pointing the way to an unorthodox solution to the mind–body problem.

At a very general level, Bohm endorsed a vision that connects an underlying reality (active information) with mind:

…reality can be considered as in essence a set of forms in an underlying universal movement or process…Thus, the way could be opened for a world view in which consciousness and reality would not be fragmented from each other.

([36], p. xiv)

More directly, Bohm suggested that “the particles of physics have certain primitive mind-like qualities” ([48], p. 272). We must always recall that for Bohm “particles” are not anything like tiny, individual entities. In the quoted philosophical article, he is writing to be understood by a wider and non-scientific, or at least non-physicist, audience. This audacious proposal integrates well with the metaphysical viewpoint we have been developing: mentality possesses the intrinsic semantics needed for active information and active information’s non-local universal presence provides support for the doctrine of holism.

The idea that mental features are a fundamental and ubiquitous feature of the world is the ancient doctrine of panpsychism. It has seen a remarkable revival in recent philosophical work (see, e.g., [49,50,51,52]). The general metaphysical outlook that places the mental as the intrinsic ground of the structural relations studied by science provides a viewpoint that integrates mind and the physical world, which leaves the physical world causally complete, avoiding outside influences distorting the laws of nature, but nonetheless provides a role for mind in the world. We can see Bohm as a kind of pioneer for this rebirth (The Bohmian approach to the mind–body problem and panpsychism is explored in depth in [53]; I have tried to explore the connection to Russellian Monism in [54]).

I will conclude with one more puzzle. The most intractable aspect of the mind–body problem is the problem of understanding consciousness. Although the identification of mentality with consciousness was philosophical orthodoxy for centuries, in modern times it has been generally accepted that mentality does not automatically imply consciousness. Bohm would seem to accept this. After the above quoted endorsement of the mentality of fundamental physical entities, he quickly goes on to add that, “of course, they do not have consciousness” ([48], p. 272).

This raises a question that has almost as many answers as there are those who ask it: what is consciousness? We can to some extent cut the complexity of this question by focusing on two basic conceptions of consciousness, call them the “thick” and the “thin” conceptions of consciousness. The thick conception is one that sees consciousness as bound up with self-awareness, or a reflective appreciation of our own mental lives and a palpable sense of knowing that one has awareness. Such a conception of consciousness is not uncommon and has a distinguished pedigree going back at least to Aristotle, who arguably equated consciousness with “awareness of awareness” (see [55]) and Leibniz who defined consciousness as “reflective knowledge of this [i.e., perceptual] inner state” (see [56]). There is evidence that Bohm too subscribed to a thick conception of consciousness. For example, he characterizes “conscious awareness” in terms of “attention, sensitivity to incoherence, all sorts of subtle feeling and thoughts and creative imagination as well as much more” ([42], p. 300). It would indeed be strange to assign all these mental functions to the lowly electron!

But there is also a thin conception of consciousness. Most think that animals can feel things such as pain and pleasure, though there is much disagreement about how far bare sentience is spread throughout nature. Nonetheless, it seems clear that primitive feelings occur without such higher functions as self-reflection or creative imagination. But feeling pain is a kind of consciousness. This thin conception is what Thomas Nagel [57] was trying to get at when he pointed out that there is “something it is like” to be an experiencing creature (famously, a bat). This basic sort of consciousness is the essence of the difficulty we have integrating consciousness into a physicalist metaphysics, because how could subjective experience arise from entirely non-experiential constituents? This is in essence David Chalmers’ famous “hard problem of consciousness” (see [58]). Given a world of physical entities entirely lacking any subjective aspect, how could the intrinsic subjectivity of conscious experience ever arise in the world?

The seeming intractability of the problem of consciousness suggests that it is not a phenomena amenable to direct scientific understanding. We can investigate the links between consciousness and physical processes, most especially of course those of the brain (though we do not know whether non-neural substrates, such as make up digital computers, are possible). However, these linkages will not reveal what consciousness is or how it arises. Bohm’s view offers a novel explanation for both the elusiveness of consciousness when examined from the ordinary scientific standpoint and offers a place for consciousness within the natural world.

It seems fairly easy to imagine that basic sentience comes in degrees of complexity, ranging down to extremely simple forms that would be little more than the merest spark of feeling. Although intellectually challenging and, to many, intuitively implausible, it is not so hard to assign such forms to the fundamental physical entities. This will be the basic case of intrinsic semantically significant active information. Presumably then, more complex forms of consciousness will emerge via some process of increasing physical complexity of structure. Bohm and Hiley have some remarks along these line in ([42], pp. 381 ff.). How exactly this kind of “mental chemistry” would work is of course mysterious, but the idea is not incoherent. If we take on board a thin conception of consciousness, we can perhaps equate it with the primitive mind-like qualities, which Bohm assigned to the foundation of the world. We would then have the outline of a complete, and anti-reductionist, solution to the mind–body problem.

In the end, Bohm’s metaphysics is about as far from that of the Newtonian classical metaphysical picture of the world as one could get. It is highly speculative and audacious. However, it appears to hold the promise of a new view of nature that integrates consciousness into the world, which science studies in a way that does not presume to dictate how science ought to proceed, nor does it suggest that mind or consciousness in any way “interferes” with natural law. At the same time, the view does not attempt to reduce or eliminate consciousness but rather offers it a place in the world as an irreducible fundamental feature of it. Overall, it is an inspiring and even exhilarating combination of philosophical and scientific metaphysics.
